# Ketogenic diet in the treatment of epilepsy in children under the age of 2 years: study protocol for a randomised controlled trial

**DOI:** 10.1186/s13063-017-1918-3

**Published:** 2017-04-26

**Authors:** Siobhan Titre-Johnson, Natasha Schoeler, Christin Eltze, Ruth Williams, Katharina Vezyroglou, Helen McCullagh, Nick Freemantle, Simon Heales, Rachel Kneen, Louise Marston, Tim Martland, Irwin Nazareth, Elizabeth Neal, Andrew Lux, Alasdair Parker, Shakti Agrawal, Penny Fallon, J. Helen Cross

**Affiliations:** 1 UCL Great Ormond Street Institute of Child Health, London, UK; 2 Great Ormond Street Hospital, London, UK; 3 Evelina London Children’s Hospital, London, UK; 4 Leeds Teaching Hospital NHS Trust, Leeds, UK; 5 PRIMENT Clinical Trials Unit, UCL, London, UK; 6 UCL Great Ormond Street Institute of Child Health and Great Ormond Street Hospital for Children NHS Foundation Trust, London, UK; 7 Alder Hey Children’s Hospital, Liverpool, UK; 8 PRIMENT Clinical Trials Unit, Department of Primary Care and Population Health, UCL, London, UK; 9 Royal Manchester Children’s Hospital, Manchester, UK; 10 Matthew’s Friends Clinics, Lingfield, Surrey, UK; 11 University of Bristol, Bristol, UK; 12 Addenbrooke’s NHS Trust, Cambridge, UK; 13 Birmingham Children’s Hospital, Birmingham, UK; 14 St. George’s University Hospitals, London, UK

**Keywords:** Ketogenic diet, Epilepsy, Randomised controlled trial, Infants

## Abstract

**Background:**

The incidence of epilepsy is greatest in the first 2 years of life, an age group where there is generally a poor prognosis for both seizure control and neurodevelopmental outcome. Early control of seizures can be associated with better developmental outcome but many of the epilepsies presenting in infancy are poorly responsive to antiepileptic medication. The ketogenic diet (KD) is a high-fat, low-carbohydrate diet designed to mimic the effects of starvation on the body. Dietary fat is converted into ketones in the body and used as an energy source by the brain. The KD has been shown to be successful in controlling seizures in many observational studies, and in two randomised controlled trials (RCTs) in older children. However, little evidence is available in the very young.

**Methods/design:**

An open-label RCT where eligible children (age 3 months to 2 years with epilepsy who have failed two antiepileptic drugs (AEDs)) undergo baseline assessment, including medical and seizure history. Participants then start an observation period (7 or 14 days) with documentation of seizure frequency. Randomisation will occur on day 8 or day 15 to receive the KD or a further AED; the allocated treatment will commence on day 15, with instruction and training. A second assessment (4 weeks after start of treatment) will include a clinical review and tolerability questionnaire (modified Hague Scale of Side Effects – for those allocated to the KD group). Assessments will be repeated at 8 weeks after the start of treatment including biochemical investigations, after which, according to patient response, KD (diet group) or AED (standard AED group) will then be continued or changed. Those in the AED group who have failed to achieve seizure control at the 8-week assessment will then be offered KD outside the context of the trial. Those in the KD arm who fail to achieve seizure control will be changed to standard clinical management. All patients will be followed up for 12 months from randomisation for retention, seizure outcome, quality of life and neurodevelopmental status.

**Discussion:**

The slow rate of recruitment is an ongoing practical issue. There is a limitation to the number of eligible patients compared to what was predicted, mainly due to the nature of this patient group. After a substantial amendment to widen inclusion criteria and reduce the baseline period to 7 days for patients with a high seizure burden, the rate of recruitment steadily increased. A number of operational concerns regarding dietetic time were also highlighted impacting on the recruitment rate. However, the combination of a low dropout rate and the opening of further centres, the trial should successfully meet the final recruitment target. All nine centres are now recruiting and we hope to open further centres within the UK.

**Trial registration:**

ClinicalTrials.gov, identifier: NCT02205931. Registered on 16 December 2013.

**Electronic supplementary material:**

The online version of this article (doi:10.1186/s13063-017-1918-3) contains supplementary material, which is available to authorized users.

## Background

Epilepsy is a condition whereby individuals are prone to recurrent epileptic seizures, a change in behaviour or movement that is the direct result of a primary change in the electrical activity in the brain. It is not a single condition – there are many different underlying causes and, more accurately, they should be referred to as the epilepsies. Up to 65% of individuals with epilepsy will have seizures controlled with antiepileptic drugs (AEDs) or enter spontaneous remission in their lifetime [1]. However, this leaves 35% who will continue with seizures despite treatment. Standard first-line management of an individual presenting with epilepsy is antiepileptic medication, decided on the basis of the type of epilepsy. Although guidelines exist on which drug to use, management is still based on a ‘trial and error’ approach [2]. When the type of epilepsy or seizure is unclear, it can be difficult to optimise treatment at the outset.

The incidence of epilepsy is greatest in the first 2 years of life (56–88/100,000 children/year), [3] a population who remain most at risk for neurodevelopmental compromise in the longer term. Early control of seizures is associated with better developmental outcome [4] but, unfortunately, many of the epilepsies presenting in infancy are associated with a poor prognosis for seizure control [5, 6]. Little evidence is available with regard to effective treatments and, even where seizure freedom is achieved, this is unlikely to be sustained long term [7]. This group of children place a large burden on health services, with a need for regular clinical review and ongoing medication, as well as clinical and therapy support. This is especially true for those who remain resistant to medication, this group being amongst the most costly for medical and care services long term. It is, therefore, imperative that all other treatment options are explored as early as possible [8].

The epilepsies in this age group also remain poorly defined entities; very few can be classified into an epilepsy syndrome and diagnosis of underlying cause remains difficult. Over 50% of infants presenting with seizures will have infantile spasms [3]. This affects approximately 1 in 2000 infants. First-line treatment options (corticosteroids or vigabatrin) lead to seizure freedom in up to 70% of cases [9] but side effects limit their duration of use and relapse rates are substantial (40%) [10]. Further, those who fail these treatments are limited in their treatment options. Of the remaining types of epilepsy, the majority are resistant to medication. There is little evidence on which to base our decisions on specific AED use in this young age group. Epidemiological data have shown this group to be the least likely to achieve longer-term remission of up to 2 years [11].

The ketogenic diet (KD) is a high-fat, low-carbohydrate diet designed to mimic the effects of starvation on the body. The main energy intake is fat, which is converted to ketones in the body and used as an energy source. The classical KD is based on an intake of long-chain fat, usually in a ratio of 3 or 4 g of fat to 1 g of carbohydrate and protein. The medium-chain triglyceride (MCT) KD utilises MCT fat, which generates more ketones per calorie compared to long-chain fat, theoretically allowing greater dietary intake of carbohydrate. More liberal diets have also been utilised, including the Modified Atkins Diet and low-glycaemic-index treatment, which are high in fat, low in carbohydrate but with unlimited protein [12, 13].

The KD has been shown to be successful in controlling seizures in many observational studies [14, 15]. However, there is limited evidence examining efficacy against no change or alternative treatments from randomised controlled trials (RCTs) [16]. The first RCT of the KD to demonstrate effectiveness in children aged 2–16 years was published in 2008 [17]. In this trial, 145 children aged 2–16 years, who had failed at least two AEDs and had at least seven seizures weekly, were randomised to receive a KD, either immediately or after a 3-month delay with no additional treatment changes (the latter being the control group). After 3 months, the mean percentage of baseline seizures (on an intention-to-treat analysis) was significantly lower in the diet group (62%) than in controls (137%, *p* < 0.0001). Twenty-eight (38%) of the diet group had greater than 50% seizure reduction, compared to four (6%) controls (*p* < 0.0001). One further RCT of similar design and in the same age group has been reported demonstrating efficacy of the Modified Atkins Diet to a similar degree [13]. There has been no RCT assessing treatment with the KD in children under the age of 2 years.

Open-label, observational evidence indicates that the KD could be particularly effective in younger children. Open-label studies have suggested it to be an effective and well-tolerated treatment for infants [12, 18–26]. One study reported significant improvements in infantile spasms and fewer side effects when the diet was used as an alternative first-line therapy to adrenocorticotrophic hormone (ACTH) [27].

The mechanism of action of the KD is not yet known. Recent evidence suggests that medium-chain fatty acids, more specifically decanoic acid, may have a specific role in its antiepileptic effect [28–30]. These data raise the possibility that C10 alone has the ability to mimic aspects of the KD. Whether this has a role in a possibly enhanced action of the KD in infancy should be determined and the biochemical basis for effectiveness identified [31, 32].

The KD is a high-resource treatment, requiring patient-specific calculation and regular input from a specialist paediatrician and dietitian, with close monitoring thereafter. It also requires diligence on the part of the families. It is also not without side effects. It is imperative that the effectiveness and safety of the KD in this very young age group is now studied in a well-designed clinical trial.

### Study objectives

The primary objective is to assess the effectiveness of the KD compared to AEDs in the treatment of infants with epilepsy aged 3 months to 2 years of age who continue to have seizures despite previous trials of two AEDs. The secondary objectives are to determine tolerability of the KD relative to standard AEDs, adherence to treatment over time, the effect on quality of life and neurobehavioural progress. Further, we aim to estimate whether the presence of medium-chain fatty acids in the context of use of the KD is associated with seizure control.

## Methods/design

The project proposed is an open-label, randomised controlled, multicentre clinical trial of children aged 3 months to 2 years of age with epilepsy who have failed to respond to two or more pharmacological treatments (AED or corticosteroids), comparing KD to further AED treatment. The study will be conducted in two phases: first, we will carry out a pilot phase in two centres. If the pilot study recruits successfully, we will proceed to the full trial in a further seven centres. See Additional file 1 for a schematic of the trial design.

Eligible children will be consented via their parents. They will undergo baseline assessment including medical and seizure history, neurological and anthropometric examination, administration of quality of life (Infant and Toddler Quality of Life Questionnaire, [33]) and developmental (Vineland Adaptive Behaviour Questionnaire, [34]) questionnaires and biochemical investigations. They will then start a 2-week observation period with documentation of seizure frequency. If the child is prone to particularly frequent seizures in excess of two per day, then a minimum baseline period of 1 week would be considered sufficient. Food diaries required for diet calculation will be returned by post from all enrolled infants 1 week (or sooner for those with an unstable clinical condition) into the observation period. Standardised seizure records will be kept during the observation period and throughout the trial. Randomisation will be conducted using an Internet randomisation system provided by Sealed Envelope™ (Sealed Envelope Ltd.). Randomisation will occur on day 8 or day 15 for participants to receive the KD or a further AED; the allocated treatment will commence following randomisation, with instruction and training.

The randomisation schedule will be independently generated and held by Sealed Envelope™. Allocations will be released by email to the coordinating centres once the investigator or research nurse has entered eligible participant information into the web-based randomisation service. Participants will be allocated to either the KD or further AED arm using a simple, concealed, randomisation method. Randomisation will aim to achieve 92 in the KD group versus 68 participants in the further AED arm (see calculation in ‘Sample size’ section). An Enrollment Log will be maintained to keep records of the screened and randomised patients at each site. Withdrawn patients will not be replaced and replacement numbers will not be issued. Whilst it will not be possible to blind participants to their treatment allocation, efforts will be made to minimise expectation bias by emphasising in the trial literature that the evidence supporting the KD for seizure control is currently limited.

**Fig. 1:**
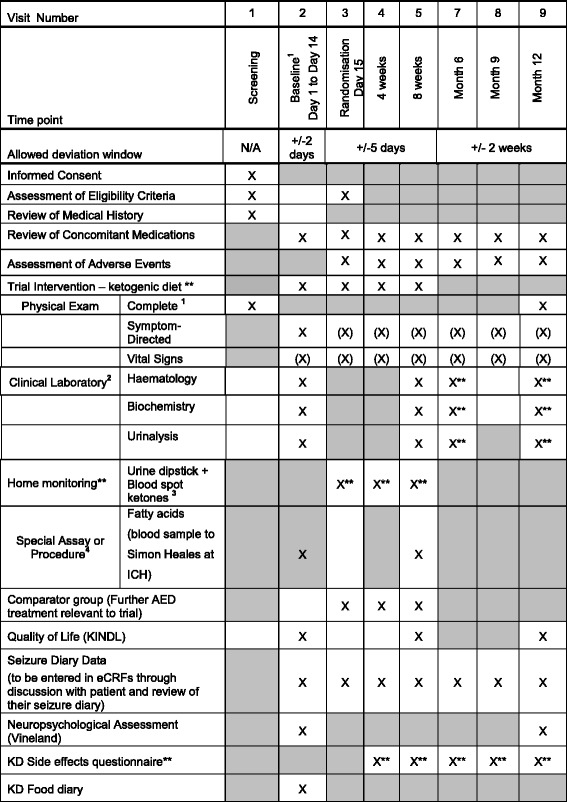
Standard Protocol Items: Recommendations for Interventional Trials (SPIRIT) figure; schedule of enrollment, interventions and assessments. *At baseline, all procedures should be done before randomisation. **Ketogenic diet group only. (X) – As indicated/appropriate. ^1^Complete physical includes weight, length, head circumference, general examination. ^2^Tests to be done: haematology – full blood count (FBC); biochemistry – liver function tests, renal function tests, calcium, urate, glucose, phosphate, vitamin D, selenium, zinc, cholesterol, carnitine profile and beta-hydroxybutyrate; urinalysis – organic acids, urine calcium and creatinine ratio. Results must be received prior to randomisation. ^3^Home monitoring urine dipstick and blood spot ketones done twice a day and recorded in Seizure Diary (only KD arm). ^4^Special assay or procedure – blood sample to be analysed by Simon Heales at ICH

A second assessment (4 weeks after start of treatment) will include clinical review and a tolerability questionnaire (modified Hague Scale of Side Effects – see Additional file 2). Assessments will be repeated at 8 weeks after the start of treatment including clinical review, administration of the Infant and Toddler Quality of Life Questionnaire, the tolerability questionnaire, and biochemical investigations. After the 8-week assessment, according to patient’s clinical response to treatment with regards seizure outcome and tolerability, the KD (diet group) or AED (standard AED group) will then be continued or changed. Those in the AED group who have failed to achieve seizure control at the 8-week assessment will then be offered the KD outside the context of the trial. Those on the KD who have failed to achieve seizure improvement at the 8-week assessment will continue with medical management, as per clinician decision. All participants will be followed up for 12 months following randomisation for retention, seizure outcome and neurodevelopmental status. See Fig. 1 (Standard Protocol Items: Recommendations for Interventional Trials (SPIRIT) figure: schedule of enrollment, interventions and assessments) and Additional file 3 for the SPIRIT 2013 Checklist (recommended items to address in a clinical trial protocol and related documents).

At each of the centres, the paediatric neurologist will work with a dietitian for KD implementation. Treatment will be in accordance with the KD Intervention Manual, which will be agreed with reference to a standard text [35] and discussed with the project management team at the outset during the initial workshop to enable standardisation of treatment between different centres. There will be an AED consensus flowchart (Additional file 4) for guidance regarding management of the participant’s epilepsy, written following an initial workshop with paediatric neurologists from all nine centres; this flowchart will be used to create a standardised manual (AED Consensus Document).

### Selection of participants

Children aged 3 months to 2 years with an established diagnosis of epilepsy, who continue to have epileptic seizures despite treatment with two AEDs, will be screened for entry into the trial. Recruitment will be from hospital-based paediatric neurology centres, with the additional involvement of a user group, Matthew’s Friends Charity, an organisation set up to raise awareness and availability of the KD in the UK, and which now also supports clinics implementing the KD. Many, if not all of the suitable patients will already be under the care of tertiary paediatric neurology centres according to National Guidelines (https://www.nice.org.uk/guidance/cg137).

#### Inclusion criteria

1. Age between 3 months and 24 months of age (not beyond second birthday at baseline)
2. Diagnosis of epilepsy confirmed
3. Seizure frequency greater than or equal to four seizures/week on average in the baseline period
4. Failed response to previous trial of two antiepileptic drugs. In the case of infantile spasms, this could include a trial of corticosteroids
5. Children with written informed consent from a parent/guardian

#### Exclusion criteria

1. Continues on corticosteroids less than 2 weeks prior to randomisation
2. Metabolic disease contraindicating use of the KD, e.g. pyruvate carboxylase deficiency, medium-chain acyl-CoA dehydrogenase (MCAD) deficiency from previous medical investigation and screening at baseline
3. Progressive neurological disease
4. Severe gastroesophageal reflux
5. Previous treatment with the KD
6. Concurrent participation in another clinical trial of an investigational medicinal product (IMP)
7. Patients who are prescribed AEDs not listed in the trial IMPs
8. Patients who have a listed contraindication as per the Summary of Product Characteristics (SmPC) to any of the AEDs listed in the trial IMPs

### Sample size

For the primary outcome variable, based on data from Neal et al., [17], we used mean percentage change in seizures from baseline of 62% (SD 45) in the KD group, assuming a change of 90% in the control group (SD 50) (100 = no change in frequency of seizures from baseline) at 90% power and 5% significance. This gives a sample size of 61 in each group (122 in total). Accounting for a 10% dropout rate, gives 68 in each group (136 in total), and inflation for a therapist effect (dietitian) to one group, assuming nine centres, with an average cluster size of eight and an intracluster correlation coefficient (ICC) of 0.05, the inflation factor is 1.35, giving 92 in the KD group and 68 in the control group (160 in total). If dropout was 20%, this sample size would still have 86% power.

#### Treatment procedures

##### Trial arm 1

Classical ketogenic diet (KD arm)

The experimental intervention will be an 8-week trial of KD therapy. A KD intervention manual will be created and provided to sites to ensure consistency of the KD implementation across centres. The manual includes basic instructions on how to calculate the classical KD and advice regarding diet implementation such as supplementation, tube feeding, breastfeeding, weaning and fine-tuning the diet. Children allocated to KD therapy will have their diets individually calculated by a paediatric dietitian with consideration of daily calorie requirements, adequate protein intake for growth and vitamin and mineral supplementation. All diets will be implemented according to a classical KD protocol, i.e. based on a ratio of fat to carbohydrate and protein that will usually be between 2:1 and 4:1. In order to achieve a state of ketosis, meal plans have to be accurately calculated for each child individually and recorded in the patient’s medical notes. Breastfeeding can be continued on a KD, in combination with a ketogenic feed which will be given in a prescribed amount before each breastfeed. If breast milk is expressed, this can be mixed with the ketogenic feed to the correct macronutrient ratio. Infants on a KD can be weaned as per Department of Health guidelines [36], with advice given on how to adapt standard weaning foods by addition of extra fat.

Hospital stays will be determined by the clinical team at the treating centre, utilising the ‘Trial Intervention Manual’. A non-fasting initiation protocol will be used for all children. Parents or carers will attend a teaching session prior to diet commencement, including how to manage possible early side effects, such as excess ketosis and hypoglycaemia. Teaching of families in the KD arm will occur following randomisation and prior to starting the diet.

The KD to be implemented will be the classical KD, aiming for at least a 3:1 ratio (fat to carbohydrate and protein). This will be implemented according to a standard text [35]. Further, an initial workshop of dietitians will be led by Elizabeth Neal (co-principal investigator (PI)) in order to ensure consistency of implementation. Cross-site consistency of KD implementation will be monitored after the 8-week and 12-month visits by the dietetic assistant. Details to be monitored include the calculation of energy prescriptions, protein intake, teaching sessions, initiation regimes, supplementation and ketone levels. Monitoring Discrepancy Forms will be created and the Protocol Deviation Log completed, if appropriate.

##### Trial arm 2

Further antiepileptic drugs (AED arm)

The control intervention will be drug therapy with the most appropriate further AED for a particular child, depending on their presenting seizures, epilepsy syndrome and previous drugs used. This will be chosen by the expert clinician responsible for management of the participant’s epilepsy. Paediatric neurologists will meet at an initial workshop to discuss clinical practice with the aim of forming the basis of a consensus protocol to ensure the consistency of AED treatments delivered. The list of agreed drugs that may be utilised are described in Additional file 5. These are considered IMPs in this trial, irrespective of which arm of the trial the patient is randomised to. This is a pragmatic trial that uses authorised medicinal products for epilepsy within the European Economic Area (EEA). Although the majority of these IMPs are not licensed for paediatric use, or for use in this age group, they are used in routine care as part of established clinical practice. Patients who are prescribed products with no marketing authorisation (‘specials’) for epilepsy or AEDs not listed in the below table will not be eligible for this trial. The KD is not classed as a medicinal product and is not included in Additional file 5. The dietetic assistant will monitor cross-site consistency of IMP prescription according to the protocol.

A discussion about diet and healthy eating will be also be undertaken with families of infants randomised to the AED arm at the randomisation visit. If the participant is already under local dietetic support, it should be ensured that this monitoring continues. If the participant does not have local dietetic support but this is deemed necessary by the ketogenic dietitian, an appropriate referral should be made by the clinician. Otherwise, a very brief discussion about general infant or toddler nutrition will be had, including details such as promotion of breastfeeding, age-appropriate texture progression for weaning, food groups and the important of iron-rich foods.

### Statistical analysis

Analysis will be done by intention-to-treat. Baseline characteristics of participants in the control and intervention arms will be summarised. The primary outcome will be seizure count in the final 2 weeks of the intervention period and in the baseline assessment period. Data will be analysed using a Poisson mixed model to account for clustering by centre (synonymous with therapist). The randomised allocation will be entered into the model as a fixed effect as will an indicator of time point (baseline or end of study), whilst the centre will be included as a random effect. Analysis of secondary outcomes (those seizure free and responders) will be analysed using random-effects logistic models – centre being the random effects and randomised group a fixed effect. The process outcomes relating to tolerability and medium-chain fatty acids in the KD group will be analysed using random-effects modelling. Therapist effects will be investigated further in supportive analyses [7, 37].

### Discontinuation/withdrawal of participants and ‘stopping rules’

Participants will be withdrawn from the treatment prior to 8 weeks should there be over 50% increase in seizure frequency from baseline or if side effects, such as diarrhoea or constipation, are not resolved by dietary manipulation or medication. Withdrawn patients will not be replaced, but will have scheduled follow-up assessments.

### Deliverability and feasibility

An initial pilot study involving two centres over 12 months will aim to recruit approximately 20% of the total sample required: 35 participants over 12 months.

The pilot study will run in two centres in London: Great Ormond Street Hospital for Children and Evelina London Children’s Hospital over the initial 12 months. We will aim to assess approximately 50 eligible patients in this time frame. Additionally, adverse events (AEs) will be monitored and data on safety will be reviewed. We will progress to the full study if the following are achieved:

1. Achieving a 60% recruitment rate; 30 families agreeing to randomisation
2. No more than 10 (29%) failing to complete the 8-week trial period

The main study will proceed to include recruitment from the further seven centres should the above criteria be met. Recruitment will be expected at a rate of 28/year from GOSH, and approximately 7/year from the remaining seven centres (total 84/year). This enables a completed primary outcome in the desired 160 children.

### Outcomes

The primary outcome will be the number of seizures experienced during weeks 6–8 compared to the number of seizures in the baseline period.

Secondary outcomes will include (at 8 weeks):

- Number of children seizure free
- Responder rate, defined as the number showing more than a 50% improvement in seizure frequency compared to baseline (taken as the mean daily seizure frequency over the observation baseline period immediately preceding the 8-week review)
- Tolerance to KD as assessed by side effect questionnaire and blood results
- Relationship between medium-chain fatty acids and seizure control

Secondary outcomes will also include (at 12 months):

- Retention on treatment (the number of participants who remain on the KD, or the prescribed AED, at 12 months)
- Quality of life (as measured by the Infant Toddler Quality of Life Questionnaire)
- Neurodevelopmental outcome (as measured by the Vineland Adaptive Behaviour Scales)

Plasma profiles of medium-chain fatty acids will be evaluated at baseline and at 8 weeks. Assessment of mitochondrial function (respiratory-chain enzymes) and enrichment (citrate synthase) will be determined in white cells and platelets. The effect of specific ratios of medium-chain fatty acids, to mimic patient plasma profiles, upon neuronal mitochondrial function/enrichment (biochemical plus electron microscopy studies) will be documented. Additionally, such fatty acid profiles will be studied, with regards to antiepileptic effect, in an established in vitro (hippocampal slice) model.

## Discussion

We summarise here the protocol of an open-label RCT, designed to evaluate the effectiveness of the KD in children with epilepsy under the age of 2 years who have failed two AEDs, compared to standard AED treatment. We hypothesise that the KD is more effective in reducing seizure frequency in infants (age 3 months to 2 years) with epilepsy who have failed to respond to two or more pharmacological agents (AED or corticosteroids) compared to conventional management with an AED.

There is genuine equipoise between the two treatment groups. There is little evidence on the effectiveness of treatments in children with epilepsy under 2 years of age, in particular comparative evidence. The most common seizure type to present in infancy is infantile spasms, for which there is evidence on first-line treatment [9]; there is no RCT-derived evidence on which treatment to use should either steroids or vigabatrin fail. Further, there is little evidence in other seizure types or syndromes in this age group. There is, therefore, a need to determine whether KD treatment or standard drug treatment should be utilised early in the natural history of the condition. Information on the effectiveness of the KD in the treatment of younger children is limited to the results of open-label, non-randomised and non-prospective studies. Further, numbers are small, children are reported only at the severe end of the spectrum and, in some, the diet is trialled in preference to conventional antiepileptic medication known to be beneficial [27]. The results from the trial in the older children [17], although still showing the diet to be effective, did not show this to the same degree as in the reported open studies. It is important to determine the place of the KD in this younger group of patients, both with regard to effectiveness and safety, considering the resource required and vulnerability of the group.

Some concern could be expressed about the duration of the trial at only 8 weeks to primary outcome; many studies utilise a 3-month assessment period, and the KD can take a while to introduce and fine tune. In the group of children to be assessed, namely children under the age of 2 years, many epilepsy syndromes are characterised by a high frequency of seizures. Further, many AEDs have a high rate of clearance in this age group. Indeed, a relatively high seizure frequency may preclude recruitment in view of the concern about a 2-week baseline. Our pilot data from previous trials and continuing work in our KD research clinics strongly suggest that seizure response to either the KD or usual AEDs is determined in the infant population by 4 weeks. Previous AED studies in this age group have also utilised a 1-day to 4-week titration period and a 4-day to 4-week stabilisation period [38–40]. The balance is between the likely time required to determine failure of the AED and the time required to establish a child on the KD. Eight weeks allows a 4–6 week titration for establishing the KD or an AED, with at least 2 weeks’ stability for seizure assessment.

### Trial Management Group

#### Membership

The committee will be made up of the following:

- Chief investigator
- Trial manager
- Principle investigators
- Trial statisticians

#### Terms of reference

- The Trial Management Group (TMG) has been established by the chief investigator (CI) as part of the trial design to monitor the conduct and progress of the trial
- The TMG will also ensure that the researchers have access to documentation necessary for the conduct of the trial and monitor data to identify unusual patterns
- Monthly update of AEs occurring at each site will be reviewed. If unexpected events occur in either of the arms, these are to be reported to the sponsor
- The TMG will assess and confirm the decision to progress to the full trial from pilot study completion at two centres, as per protocol
- The TMG will meet monthly at the start of the study and then quarterly on completion of recruitment
- The quorum for the TMG will be four members. This should include the chief investigator, two members plus the trial statistician
- The TMG meetings will be minuted and a copy of the minutes will be held in the TMG. A copy signed by the CI will be sent to the sponsor for oversight (or the CI will be included in the email copy to the sponsor)

### Trial Steering Committee

#### Membership

The Committee will be made up of the following:

- Independent chair
- Chief investigator
- Clinical trial manager
- Two parent representatives
- Principal investigators
- Two independent members
- UCL PRIMENT Clinical Trials Unit (CTU) representative
- Consumer representative
- UCL representative
- NIHR representative

#### Terms of reference

- The Trial Steering Committee (TSC) has been established by the CI as part of the trial design, to assess at intervals the progress of the trial, the safety data and the critical efficacy endpoints
- The TSC will recommend to the sponsor whether to continue, amend or stop the trial. The TSC will review the AE logs and Case Report Forms (CRFs) in line with the approved protocol
- The TSC will meet twice a year to review the safety data and efficacy endpoints captured in the trial database
- The quorum for the TSC will be four members. This should include the CI, two members plus a representative from the CTU
- Representatives of the trial sponsor and the trial funder will be invited to all TSC meetings
- The TSC will be responsible for approving all formal interim analyses of the data prior to external presentation or submission for publication
- The TSC meetings will be minuted and a copy of the minutes will be held in the TMF and a copy signed by the CI will be sent to the sponsor for oversight (or the CI will be included in the email copy to the sponsor)
- To protect patient confidentiality, trial participants’ identities and any identifiers, such as names and hospital numbers, will be removed

### Data Monitoring Committee

#### Membership

The Committee will be made up of the following:

- Independent clinician – chair
- Independent dietitian
- Independent statistician
- Study statistican

#### Terms of reference

- It is the only body involved in a trial that has access to the unblinded comparative data
- The role of its members is to monitor these data and make recommendations to the TSC on whether there any ethical or safety reasons why the trial should not continue
- The safety, rights and wellbeing of the trial participants are paramount
- The Data Monitoring Committee (DMC) considers the need for any interim analysis advising the TSC regarding the release of data and/or information
- The DMC may be asked by the TSC, trial sponsor or trial funder to consider data emerging from other related studies
- Membership of the DMC should be completely independent, small (three to four members) and comprise experts in the field, such as a clinician with experience in the relevant area and an expert trial statistician
- Responsibility for calling and organising DMC meetings lies with the CI, in association with the chair of the DMC. The project team should provide the DMC with a comprehensive report, the content of which should be agreed in advance by the chair of the DMC
- The DMC should meet at least annually, or more often as appropriate, and meetings should be timed so that reports can be fed into the TSC

### Trial status

The trial was initiated in the pilot phase on 1 February 2015 and is currently recruiting in the full trial phase across nine centres. We are in the process of initiating further centres as part of the next amendment. Twenty-nine patients have been randomised to date and the planned recruitment end date is July 2018.

## Additional files

Additional file 1: Schematic of trial design. (DOCX 71 kb)

Additional file 2: Treatment Side Effects Questionnaire. (DOCX 14 kb)

Additional file 3: SPIRIT 2013 Checklist: recommended items to address in a clinical trial protocol and related documents. Shows the recommended items to address in a clinical trial protocol and related documents in accordance with the SPIRIT 2013 template. This includes a template parental Consent Form, Patient Information Sheet and GP Letter. (PDF 326 kb)

Additional file 4: (AED consensus flowchart). (DOCX 44 kb)

Additional file 5: Names and descriptions of investigational medicinal products used in KIWE. (DOCX 15 kb)

## Abbreviations

AE: Adverse event
AED: Antiepileptic drug
CI: Chief investigator
CRF: Case Report Form
CTU: Clinical Trials Unit
DMC: Data Monitoring Committee
EudraCT: European Clinical Trials Database
IMP: Investigational Medicinal Product
KD: Ketogenic diet
PI: Principal investigator
PIS: Patient Information Sheet
RCT: Randomised controlled trial
SmPC: Summary of Product Characteristics
TMG: Trial Management Group
TSC: Trial Steering Committee
