# Supplementary material for: Ketogenic diet in the treatment of epilepsy in children under the age of 2 years: study protocol for a randomised controlled trial
Source: Trials. 2017 Apr 26;18:195. doi: 10.1186/s13063-017-1918-3 (PMC5406967; doi:10.1186/s13063-017-1918-3)
Supplement: Supplementary file 1 — Schematic of trial design. (DOCX 71 kb) [file 13063_2017_1918_MOESM1_ESM.docx]

**Additional file 2 - Schematic of Trial Design**

A B C F G I

Consent/Baseline

(Day 1 to Day 7 or 14)

A

B

F

G

Randomisation (Day 8 or 15

A C E J* K*

Ketogenic diet (KD)

N=92

Further AED

N=68

J*

E C D*

4 week assessment Clinical review

4 week assessment Clinical review

E, J

J*

8 week assessment – seizure outcome

8 week assessment – seizure outcome

F

C

A

A B C D* E F K*

D

B

Continue or decision to change Offered KD

Continue or decision to change

End of primary outcome

A C D*

3m review

A B C D*

6m review

A B C D* F G

H

A C D*

9m review

12m review

| **A** | **Physical exam** (complete or symptom directed) including weight, length, head circumference, general examination |
| --- | --- |
| **B** | **Clinical Laboratory** including full blood count, liver function tests, renal function, calcium, urate, glucose, phosphate, vitamin D, selenium, zinc , cholesterol, carnitine profile, beta-hydroxybutyrate and urine organic acids, urine calcium and creatinine ratio. |
| **C** | Issue/collect seizure diaries |
| **D*** | Administer side effects questionnaire |
| **E** | Trial intervention. KD or further AED comparator group |
| **F** | Infant Toddler Quality of Life Questionnaire |
| **G** | Vineland Adaptive Behavior Questionnaire |
| **H** | Seizure recording must be captured daily 28 days before 12 month review |
| **I** | Issue/collect food diary (baseline only) |
| **J*** | Home monitoring including urine dipstick and blood spot ketones |
| **K*** | Special Assay or procedure. Blood Sample to be analysed by Simon Heales at ICH (KD arm only) |

H

*KD arm only
