# Supplementary material for: Ketogenic diet in the treatment of epilepsy in children under the age of 2 years: study protocol for a randomised controlled trial
Source: Trials. 2017 Apr 26;18:195. doi: 10.1186/s13063-017-1918-3 (PMC5406967; doi:10.1186/s13063-017-1918-3)
Supplement: Supplementary file 2 — Treatment Side Effects Questionnaire. (DOCX 14 kb) [file 13063_2017_1918_MOESM2_ESM.docx]

**Additional file 3. Treatment Side Effects Questionnaire**

**Study Protocol Number:** 13/0656

**Patient Identification Number for this trial:** ……………………

**Patient Initials: ………………….**

**Centre Name:** …………………………………

**TREATMENT SIDE EFFECTS QUESTIONNAIRE**

Have you noticed any of the following side effects in your child during the past 4 weeks / 3 months / 6 months? (**please circle**)

a = yes, it is a very serious problem

b = yes, it is a moderately serious problem

c = yes, it is a mild problem

d = no *or* not applicable

1 drowsiness, sleepiness a b c d

2 sickness a b c d

3 constipation a b c d

4 diarrhoea a b c d

5 abdominal pain a b c d

6 loss of appetite a b c d

7 lack of energy a b c d

8 hyperactivity a b c d

9 hunger a b c d

10 decreased concentration a b c d

11 behavioural disturbance a b c d

……………………………….. …………….…………….

Signature Date
