# Supplementary material for: Ketogenic diet in the treatment of epilepsy in children under the age of 2 years: study protocol for a randomised controlled trial
Source: Trials. 2017 Apr 26;18:195. doi: 10.1186/s13063-017-1918-3 (PMC5406967; doi:10.1186/s13063-017-1918-3)
Supplement: Supplementary file 3 — SPIRIT 2013 Checklist: recommended items to address in a clinical trial protocol and related documents. Shows the recommended items to address in a clinical trial protocol and related documents in accordance with the SPIRIT 2013 template. This includes a template parental Consent Form, Patient Information Sheet and GP Letter. (PDF 326 kb) [file 13063_2017_1918_MOESM3_ESM.pdf]

Additional file 1-SPIRIT 2013 Checklist: Recommended items to address in a clinical trial protocol and related documents

Section/item	Item No	Description
**Administrative information**		
Title	1	Ketogenic diet in the treatment of epilepsy in children under the age of two years, KIWE
Trial registration	2a	Clinicaltrials.gov NCT02205931. Registered 16 December 2013
Protocol version	3	27th October 2015 Version 5
Funding	4	This project is funded by the Efficacy and Mechanism Evaluation (EME) Programme, an MRC and NIHR partnership (EME funding reference: 12/10/18). The EME Programme is funded by the MRC and NIHR, with contributions from the CSO in Scotland and NISCHR in Wales and the HSC R&D Division, Public Health Agency in Northern Ireland.

Roles and responsibilities	5a	Siobhan Titre-Johnson, UCL Great Ormond Street Institute of Child Health UCL Institute of Child Health (Trial Manager)<br><br>Natasha Schoeler, UCL Great Ormond Street Institute of Child Health UCL Institute of Child Health (Dietetic Assistant)<br><br>Christin Eltze, Great Ormond Street Hospital (Principal Investigator)<br><br>Ruth Williams, Evelina London Children’s Hospital (Principal Investigator)<br><br>Katharina Vezyroglou, Great Ormond Street Hospital (Co-Investigator)<br><br>Helen McCullagh, Leeds Teaching Hospital NHS Trust (Principal Investigator<br><br>Nick Freemantle, PRIMENT Clinical Trials Unit (Statistician)<br><br>Simon Heales, UCL Great Ormond Street Institute of Child Health UCL Institute of Child Health &and Great Ormond Street Hospital for Children NHS Foundation Trust (Central Laboratory)<br><br>Rachel Kneen, Alder Hey Children’s Hospital (Principal Investigator)<br><br>Louise Marston, PRIMENT Clinical Trials Unit, Department of Primary Care and Population Health, UCL (Statistician)<br><br>Tim Martland, Royal Manchester Children’s Hospital (Principal Investigator)<br><br>Irwin Nazareth, PRIMENT Clinical Trials Unit, Department of Primary Care and Population Health, UCL (Director of PRIMENT CTU)<br><br>Elizabeth Neal, Matthew’s Friends Clinics (Co-Investigator)<br><br>Andrew Lux, University of Bristol (Principal Investigator)<br><br>Alasdair Parker, Addenbrookes NHS Trust (Principal Investigator)<br><br>Shakti Agrawal, Birmingham Children’s Hospital (Principal Investigator)<br><br>Penny Fallon, St George's University Hospitals (Principal Investigator)<br><br>Judith Helen Cross, UCL Great Ormond Street Institute of Child Health and Great Ormond Street Hospital for Children NHS Trust (Chief Investigator)

5b Nimrita Verma (Sponsor’s Representative), Joint Research Office, UCL, 1st Floor Maple House, Suite A, 149 Tottenham Court Road, London, W1T 7DN
Email: n.verma@ucl.ac.uk

5c All proposed publications will be discussed with Sponsor prior to publishing other than those presented at scientific forums/meetings. Analysis will be done on an intention-to-treat model. A full statistical analysis plan will be created by PRIMENT. The sponsor will ensure that the trial protocol, patient information sheet, consent form, GP letter and submitted supporting documents have been approved by the appropriate regulatory body (MHRA in UK) and a main research ethics committee, prior to any patient recruitment. The protocol and all agreed substantial protocol amendments, will be documented and submitted for ethical and regulatory approval prior to implementation. Before the site can enrol patients into the trial, the Chief Investigator/Principal Investigator or designee must apply for NHS permission from their Trust Research & Development (R&D) and be granted written permission. It is the responsibility of the Chief Investigator/ Principal Investigator or designee at each site to ensure that all subsequent amendments gain the necessary approval. The trial is being financed by an NIHR EME programme grant for a period of 4 years.

5d The study will be supported by the UCL PRIMENT CTU. The Chief Investigator (CI) will maintain day to day responsibility for the trial working in close collaboration with the Clinical Trial Manager (CTM) to ensure that the trial is conducted, recorded and reported in accordance with the protocol, good clinical practice guidelines, and essential standard operating procedures on all aspects of trial management, quality control and data analyses required for running RCTs as documented by our Clinical Trials Unit. All investigators will have up to date Good Clinical Practice training.

A Trial Management Group (TMG) consisting of the CI, some of the co-applicants the CTM and the trial statistician will meet monthly at the start of the study and then quarterly on completion of recruitment. They will monitor the conduct and progress of the trial. The PI, CTM and statistician will monitor data to identify unusual patterns (Central Monitoring Processes). They will also ensure that the researchers have access to documentation necessary for the conduct of the trial.

The study will be overseen by an externally led Trials Steering Group (TSG) that will meet twice a year which will include two parent representatives recruited from Young Epilepsy.

We will also appoint a trial data monitoring committee (DMC) that will examine baseline data and then explore the preliminary analyses of study outcomes and adverse events conducted on the data at defined time points as determined by the Chair of the DMC. Based on predefined cut off agreed by the DMC for specified outcomes the DMC will advise the TSG on the progress of the trials.

Terms of Reference will be in place for each committee.

Introduction

Background and rationale | 6a

Epilepsy is a condition whereby individuals are prone to recurrent epileptic seizures, a change in behaviour or movement that is the direct result of a primary change in the electrical activity in the brain. It is not a single condition - there are many different underlying causes and more accurately, they should be referred to as the epilepsies. Up to 65% of individuals with epilepsy will have seizures controlled with antiepileptic drugs (AED) or enter spontaneous remission in their lifetime. However, this leaves 35% who will continue with seizures despite treatment. Standard first line management of an individual presenting with epilepsy is antiepileptic medication, decided on the basis of the type of epilepsy. Although guidelines exist on which drug to use, management is still based on a ‘trial and error’ approach. When the type of epilepsy or seizure is unclear, it can be difficult to optimise treatment at the outset.

The incidence of epilepsy is greatest in the first two years of life (56-88/100,000 children/year), a population who remain most at risk for neurodevelopmental compromise in the longer term. Early control of seizures is associated with better developmental outcome but, unfortunately, many of the epilepsies presenting in infancy are associated with a poor prognosis for seizure control. Little evidence is available with regard to effective treatments and, even where seizure freedom is achieved, this is unlikely to be sustained long-term. This group of children place a large burden on health services, with a need for regular clinical review and ongoing medication, as well as clinical and therapy support. This is especially true for those who remain resistant to medication, this group being amongst the most costly for medical and care services long-term. It is therefore imperative that all other treatment options are explored as early as possible.

The ketogenic diet (KD) is a high-fat, low-carbohydrate diet designed to mimic the effects of starvation on the body. The main energy intake is fat, which is converted to ketones in the body and used as an energy source. The KD has been shown to be successful in controlling seizures in many observational studies. However, there is limited evidence examining efficacy against no change or alternative treatments from randomised controlled trials (RCTs).The first RCT of the KD, demonstrating effectiveness in children aged 2-16 years was published in 2008. In this trial, 145 children aged 2-16 years, who had failed at least two AEDs and had at least seven seizures weekly were randomized to receive a KD, either immediately or after a 3-month delay with no additional treatment changes (the latter being the control group). After three months, the mean percentage of baseline seizures (on an intention to treat analysis) was significantly lower in the diet group (62%) than in controls (137%, P <0.0001). Twenty-eight (38%) of the diet group had greater than 50% seizure reduction, compared to four (6%) controls (p<0.0001).

The mechanism of action of the KD is not yet known. Recent evidence suggests that medium chain fatty acids, more specifically decanoic acid, may have a specific role in its antiepileptic effect. These data raise the possibility that C10 alone has the ability to mimic aspects of the KD.

	6b	The KD is a high resource treatment, requiring patient-specific calculation and regular input from a specialist paediatrician and dietitian, with close monitoring thereafter. It also requires diligence on the part of the families. It is also not without side effects. It is imperative that the effectiveness and safety of the KD in this very young age group is now studied in a well-designed clinical trial.
Objectives	7	The primary objective is to assess the effectiveness of the KD compared to AEDs in the treatment of infants with epilepsy aged three months to two years of age who continue to have seizures despite previous trials of two AEDs. The secondary objectives are to determine tolerability of the KD relative to standard AEDs, adherence to treatment over time, the effect on quality of life and neurobehavioural progress. Further, we aim to estimate whether the presence of medium chain fatty acids in the context of use of the KD is associated with seizure control.
Trial design	8	An open label RCT where eligible children (age three months to two years with epilepsy who have failed two antiepileptic drugs [AEDs]) undergo baseline assessment, including medical and seizure history. Participants then start an observation period (7 or 14 days) with documentation of seizure frequency. Randomisation will occur on Day 8 or Day 15 to receive the KD or a further AED; the allocated treatment will commence on day 15, with instruction and training. A second assessment (four weeks after start of treatment) will include clinical review and tolerability questionnaire (modified Hague scale of side effects – for those allocated to the KD group). Assessments will be repeated at eight weeks after the start of treatment including biochemical investigations, after which, according to patient response, KD (diet group) or AED (standard AED group) will then be continued or changed. Those in the AED group who have failed to achieve seizure control at the eight week assessment will then be offered KD outside the context of the trial. Those in the KD arm who fail to achieve seizure control will be changed to standard clinical management. All patients will be followed up for 12 months from randomisation for retention, seizure outcome, quality of life and neurodevelopmental status.

Methods: Participants, interventions, and outcomes

Study setting	9	Recruitment will be from hospital-based paediatric neurology centres, with the additional involvement of a user group, Matthews Friends Charity, an organisation set up to raise awareness and availability of the KD in the UK, and which now also supports clinics implementing the KD. Many, if not all of the suitable patients will already be under the care of tertiary paediatric neurology centres according to National Guidelines (www.nice.org.uk/cg137).

Eligibility criteria	10	**Inclusion:** Age between three months and 24 months of age (not beyond second birthday at baseline, diagnosis of epilepsy confirmed, seizure frequency greater than or equal to four seizures/ week on average in the baseline period, failed response to previous trial of two antiepileptic drugs. In the case of infantile spasms, this could include a trial of corticosteroid, Children with written informed consent from parent/guardian and exclusion criteria for participants.<br>**Exclusion:** Continues on corticosteroids less than two weeks prior to randomisation, metabolic disease contraindicating use of the KD, e.g. pyruvate carboxylase deficiency, medium-chain acyl-CoA dehydrogenase (MCAD) deficiency from previous medical investigation and screening at baseline, progressive neurological disease, severe gastroesophageal reflux, previous treatment with the KD, concurrent participation in another clinical trial of an investigational medicinal product, patients who are prescribed AEDs not listed in the trial IMPs, patients who have a listed contraindication as per the SmPC to any of the AEDs listed in the trial investigational medicinal products (IMPs).
Interventions	11a	An open label RCT where eligible children (age three months to two years with epilepsy who have failed two antiepileptic drugs [AEDs]) undergo baseline assessment, including medical and seizure history. Participants then start an observation period (7 or 14 days) with documentation of seizure frequency. Randomisation will occur on Day 8 or Day 15 to receive the KD or a further AED; the allocated treatment will commence on day 15, with instruction and training. A second assessment (four weeks after start of treatment) will include clinical review and tolerability questionnaire (modified Hague scale of side effects – for those allocated to the KD group). Assessments will be repeated at eight weeks after the start of treatment including biochemical investigations, after which, according to patient response, KD (diet group) or AED (standard AED group) will then be continued or changed. All patients will be followed up for 12 months from randomisation for retention, seizure outcome, quality of life and neurodevelopmental status.
	11b	Those in the AED group who have failed to achieve seizure control at the eight week assessment will then be offered KD outside the context of the trial. Those in the KD arm who fail to achieve seizure control will be changed to standard clinical management.

	11c	Cross-site consistency of KD implementation will be monitored after the 8-week and 12-month visits by the Dietetic Assistant. Details to be monitored include the calculation of energy prescriptions, protein intake, teaching sessions, initiation regimes, supplementation and ketone levels.<br>Treatment compliance will be captured with the use of seizure diary and food diary. Noncompliance to the Protocol study procedures will be documented by the investigator and reported to the Sponsor as agreed. Persistent noncompliance may lead the patient to be withdrawn from the study. Efficacy of the KD and further AEDs will be assessed through documentation of seizure frequency by seizure diaries completed by parents throughout, with documentation to CRF at each assessment.
Outcomes	12	The primary objective is to assess the effectiveness of the KD compared to AEDs in the treatment of infants with epilepsy aged three months to two years of age who continue to have seizures despite previous trials of two AEDs. The secondary objectives are to determine tolerability of the KD relative to standard AEDs, adherence to treatment over time, the effect on quality of life and neurobehavioural progress. Further, we aim to estimate whether the presence of medium chain fatty acids in the context of use of the KD is associated with seizure control.
Participant timeline	13	The schedule of enrolment interventions and assessments (see figure 1).
Sample size	14	Recruitment will be expected at a rate of 28/year from GOSH, and approximately 7/year from the remaining seven centres (total 84/year). This enables a completed primary outcome in the desired 160 children.
Recruitment	15	An initial pilot study involving two centres over twelve months will aim to recruit approximately 20% of the total sample required: 35 participants over 12 months. The pilot study will run in two centres in London: Great Ormond Street Hospital for Children and Evelina London Children’s Hospital over the initial 12 months. We will aim to assess approximately 50 eligible patients in this time frame. Additionally, Adverse Events (AEs) will be monitored and data on safety will be reviewed.

Methods: Assignment of interventions (for controlled trials)

Allocation:

Sequence generation	16a	The randomisation schedule will be independently generated and held by Sealed Envelope. Allocations will be released by email to the coordinating centres once the investigator or research nurse has entered eligible participant information into the web-based randomisation service. Participants will be allocated to either the KD or further AED arm using a simple, concealed, randomisation method. Randomisation will aim to achieve 92 in the KD group vs 68 participants in the further AED arm.
Allocation concealment mechanism	16b	Whilst it will not be possible to blind participants to their treatment allocation, efforts will be made to minimise expectation bias by emphasising in the trial literature that the evidence supporting the KD for seizure control is currently limited.
Implementation	16c	The randomisation schedule will be independently generated and held by Sealed Envelope and allocations released by email to the once the investigator or research nurse has entered eligible participant information into the web-based randomisation service.
Blinding (masking)	17a	Not applicable
	17b	Not applicable

Methods: Data collection, management, and analysis

Data collection methods	18a	The investigator will ensure the accuracy of all data entered in the CRFs. The delegation log will identify all those personnel with responsibilities for data collection and handling, including those who have access to the trial database. Whilst inpatient and at each outpatient visit, medical notes will form the source data for the trial and be completed as per routine practice.
	18b	Participants will be withdrawn from the treatment prior to 8 weeks should there be over 50% increase in seizure frequency from baseline, or if side effects e.g. diarrhoea, constipation are not resolved by manipulation of KD or medication. Withdrawn patients will not be replaced, but will have 12 month follow up assessment.

Methods: Monitoring

Data management	19	Data collected on paper source data will be either entered onto the electronic database by the local site or sent to the coordinating centre for data entry. These data are subjected to range and consistency checks and any queries so identified are checked against the paper records or with the relevant participating centre. There are standard existing procedures for checking discordant data on paper records with each of the participating centres if necessary.
Statistical methods	20a	Analysis will be done by intention-to-treat. Baseline characteristics of participants in the control and intervention arms will be summarised. The primary outcome will be seizure count in the final two weeks of the intervention period and in the baseline assessment period. Data will be analysed using a Poisson mixed model to account for clustering by centre (synonymous with therapist). The randomised allocation will be entered into the model as a fixed effect as will an indicator of time point (baseline or end of study), whilst the centre will be included as a random effect. Analysis of secondary outcomes (those seizure free and responders) will be analysed using random effects logistic models - centre being the random effects and randomised group a fixed effect. The process outcomes relating to tolerability and medium chain fatty acids in the KD group will be analysed using random effects modelling. Therapist effects will be investigated further in supportive analyses.

Data monitoring	21a	The Committee will be made up of the following: Independent clinician – chair, independent dietitian, independent statistician and study statistican.<br><br>It is the only body involved in a trial that has access to the unblinded comparative data. The role of its members is to monitor these data and make recommendations to the TSC on whether there any ethical or safety reasons why the trial should not continue. The safety, rights and well-being of the trial participants are paramount. The Data Monitoring Committee (DMC) considers the need for any interim analysis advising the TSC regarding the release of data and/or information. The DMC may be asked by the TSC, Trial Sponsor or Trial Funder to consider data emerging from other related studies. Membership of the DMC should be completely independent, small (3 – 4 members) and comprise experts in the field, such as a clinician with experience in the relevant area and an expert trial statistician. Responsibility for calling and organising DMC meetings lies with the Chief Investigator, in association with the Chair of the DMC. The project team should provide the DMC with a comprehensive report, the content of which should be agreed in advance by the Chair of the DMC. The DMC should meet at least annually, or more often as appropriate, and meetings should be timed so that reports can be fed into the TSC.
	21b	Analysis will be done on an intention-to-treat model. A full statistical analysis plan will be created by PRIMENT CTU, UCL. The pilot study will run in two centres in London; Great Ormond Street Hospital for Children and the Evelina Children’s Hospital in London over the initial 12 months. Baseline characteristics of participants in the control and intervention arms will be summarised. Frequencies and measures of central tendency will be calculated by randomised group to check for differences in the data.

Harms	22	All adverse events will be recorded in the medical records and CRF following randomisation, apart from parent reported seizures which will be recorded in the seizure diary. Ongoing seizures are an expected adverse event in this population and only seizures that meet the seriousness criteria will be entered on the CRF AE, The AE log will be reviewed at the TMG meetings although immediate review (within 24 hours) of SAEs will be performed by the CI/PI. If the investigator suspects that the subjects' condition has progressed faster due to the administration of the IMP, then they will record and report this as an unexpected adverse event. Clinically significant abnormalities in the results of objective tests (e.g. laboratory variables, EEG) will also be recorded as adverse events. If the results are not expected as part of disease or IMP, these will also be recorded as unexpected. All adverse events will be recorded with clinical symptoms and accompanied with a simple, brief description of the event, including dates as appropriate.
Auditing	23	All Consent forms to be 100% checked and source data verification for at least 25% of the total patient numbers to be completed by the Trial Manager/Site Monitor. Consistency between centres will be ensured by utilisation of a dietetic assistant who will visit each centre on two occasions during the course of the study to verify standard implementation of the diet and AED use as per the treatment manual written at the outset of the study.

Ethics and dissemination

Research ethics approval	24	Favourable ethical opinion was obtained from NRES Committee London - Fulham (REC reference 14/LO/1230) on the 2nd September 2014 and local R & D approval issued by Great Ormond Street Hospital for Children NHS Foundation Trust, Guy's and St Thomas' NHS Foundation Trust (to cover the Evelina London Children's Hospital) and Cambridge University Hospitals NHS Foundation Trust. HRA approval was issued on the 18th May 2016 to cover local approval within the following centres: University Hospitals Bristol NHS Foundation Trust, Birmingham Children's Hospital NHS Foundation Trust, Alder Hey Children's Hospital, Leeds Teaching Hospitals NHS Trust, St George's University Hospitals NHS Foundation Trust and Central Manchester University Hospitals Foundation Trust. Confirmation of Capacity and Capability were also carried out by the above centres and an "open to recruitment" letter issued by the sponsor, University College London (UCL).
Protocol amendments	25	The protocol and all agreed substantial protocol amendments, will be documented and submitted for ethical and regulatory approval prior to implementation. It is the responsibility of the Chief Investigator/ Principal Investigator or designee at each site to ensure that all subsequent amendments gain the necessary approval.

Consent or assent	26a	Eligible children will be consented via their parents.
	26b	All data will be handled in accordance with the UK Data Protection Act 1998. The Case Report Forms (CRFs) will not bear the subject's name or other personal identifiable data. The subject's initials, date of birth and trial identification number, will be used for identification.
Confidentiality	27	All paper source data are kept in a secure area at the coordinating centre during the course of the study and then stored by the chief investigator for 20 years.
Declaration of interests	28	JHC and SH have submitted a patent for C10, with UCL Business and Vitaflo, in the treatment of epilepsy. JHCy; JHC has received royalties for a chapter on childhood epilepsy in Brain Diseases of the Nervous System, and as editor of Paediatric Epilepsy; has received research support from the National Institute for Health and Research, the European Framework FP7, the Charles Wolfson Foundation, Action Medical Research (AMR), SPARKS and Vitaflo, is Chief Investigator (UK) for studies funded by GW Pharma, and has received honoraria paid to her department for participation in advisory boards from Zogenix, GW Pharma, Eisai, Sanofi and Nutricia. JHC is also an NIHR Senior Investigator for Paediatrics. SH has received grant funding from Vitaflo and acts as a consultant. STJ, NS, CE, RW, KV, HM, NF, RK, LM, TM, IN, EN, AL, AP, SA and PF have no competing interests.
Access to data	29	The investigator(s)/ institution(s) will permit trial-related monitoring, audits, REC review, and regulatory inspection(s), providing direct access to source data/documents. Trial participants are informed of this during the informed consent discussion. Participants will consent to provide access to their medical notes.
Ancillary and post-trial care	30	Participants may also be able to claim compensation for injury caused by participation in this clinical trial without the need to prove negligence on the part of University College London or another party. Participants who sustain injury and wish to make a claim for compensation should do so in writing in the first instance to the Chief Investigator, who will pass the claim to the Sponsor's Insurers, via the Sponsor's office.
Dissemination policy	31a	Professor Nicholas Freemantle is the trial statistician who will be responsible for all statistical aspects of the trial from design through to analysis and dissemination. All proposed publications will be discussed with Sponsor prior to publishing other than those presented at scientific forums/meetings.

	31b	STJ is the Trial Manager and PhD student on the trial. JHC is the Prince of Wales's Chair of Childhood Epilepsy, and Deputy Lead of the Developmental Neurosciences Programme at UCL-Institute of Child Health.
**Appendices**		
Informed consent materials	32	1: Informed consent form v1.0 dated 17/06/2014<br>2: Patient information sheet v2.0 dated 02/09/2015<br>3: GP letter v1.0 dated 17/06/2014
Biological specimens	33	The blood and urine samples for the secondary outcome will be processed at Local Labs. The blood samples to evaluate the plasma profiles of medium chain fatty acids and for the assessment of mitochondrial function (respiratory chain enzymes) and enrichment (citrate synthase) will be processed at the Chemical Pathology Laboratory at GOSH.

*It is strongly recommended that this checklist be read in conjunction with the SPIRIT 2013 Explanation & Elaboration for important clarification on the items. Amendments to the protocol should be tracked and dated. The SPIRIT checklist is copyrighted by the SPIRIT Group under the Creative Commons "Attribution-NonCommercial-NoDerivs 3.0 Unported" license.

Figure 1: The schedule of enrolment interventions and assessments

Visit Number		1	2	3	4	5	6	7	8	9
Time point		Screening	Baseline* Day 1 to Day 14	Randomisation Day 15	4 weeks	8 weeks	Month 3	Month 6	Month 9	Month 12
Allowed deviation window		N/A	+/-2 days	+/-2 days			+/- 1 week			
Informed Consent		X								
Assessment of Eligibility Criteria		X		X						
Review of Medical History		X								
Review of Concomitant Medications			X	X	X	X	X	X	X	X
Assessment of Adverse Events				X	X	X	X	X	X	X
Trial Intervention – ketogenic diet **			X	X	X	X				
Physical Exam	Complete [1]	X								X
	Symptom-Directed		X	(X)	(X)	(X)	(X)	(X)	(X)	(X)
	Vital Signs		(X)	(X)	(X)	(X)	(X)	(X)	(X)	(X)
Clinical Laboratory[2]	Haematology		X			X		X		X
	Biochemistry		X			X		X		X
	Urinalysis		X			X		X		X
Home monitoring **	Urine dipstick + Blood spot ketones [3]			X**	X**	X**				
Special Assay or Procedure[4]	Fatty acids (blood sample to Simon Heales at ICH)**			X**		X**				
Comparator group (Further AED treatment relevant to trial)				X	X	X				
Quality of Life (ITQOL-97)			X			X				X
Seizure Diary Data (to be entered in eCRFs through discussion with patient and review of their seizure diary)			X	X	X	X	X	X	X	X
Neuropsychological Assessment (Vineland)			X							X
KD Side effects questionnaire**					X**	X**	X**	X**	X**	X**
KD Food diary			X							

*At baseline, all procedures should be done before randomisation
**Ketogenic diet group only
(X) – As indicated/appropriate
[1] Complete physical includes weight, length, head circumference, general examination
[2]Tests to be done: Haematology – Full Blood Count (FBC) ; Biochemistry – liver function tests, renal function tests, calcium, urate, glucose, phosphate, vitamin D, selenium, zinc, cholesterol, carnitine profile and beta-hydroxybutyrate; Urinalysis – organic acids, urine calcium and creatinine ratio. Results must be received prior to randomisation.
[3] Home monitoring urine dipstick and blood spot ketones done twice a day and recorded in Seizure diary (only KD arm)

[4]Special assay or procedure – Blood sample to be analysed by Simon Heales at ICH.

Appendix 1: Informed consent form v1.0 dated 17/06/2014

PARENTAL CONSENT FORM

Study Protocol Number: 13/0656

Patient Identification Number for this trial: ……………………

Title of project: Ketogenic diet in infants with epilepsy (KIWE)

Centre Name: …………………………………

Please initial box

1. I confirm that I have read and understand the information sheet dated............................ (version...........) for the above study. I have had the opportunity to consider the information, ask questions and have had these answered satisfactorily. ☐

2. I understand that my child's participation is voluntary and that we are free to withdraw at any time without giving any reason, without his/her medical care or legal rights being affected. ☐

3. I understand that relevant sections of my child's medical notes and data collected during the study, may be looked at by individuals from the sponsor of the trial (University College London) and responsible persons authorised by the sponsor, from regulatory authorities or from the NHS Trust, where it is relevant to my child taking part in this research. I give permission for these individuals to have access to my child's records. ☐

4. I agree to my child's GP being informed of his/her participation in the study. ☐

5. I agree for ………………………………………to take part in the above study. ☐

______________________________	_______________	_______________
Name of parent & relationship with the trial subject	Date	Signature

______________________________	_______________	_______________
Name of Person taking consent (delegated by PI)	Date	Signature

When completed: 1 for participant; 1 (original) for researcher site file; 1 to be kept in medical notes.

INFORMATION SHEET FOR PARENTS/GUARDIANS

Study Title: Ketogenic Diet in Infants With Epilepsy (KIWE)

We would like to invite you and your child to take part in our research study. Before you decide we would like you to understand why the research is being done and what it would involve for you. One of our team will go through the information sheet with you and answer any questions you may have.

Please take time to read the following information carefully and ask questions if anything is not clear, or if you would like further information. Take time to decide whether or not you wish for your child to take part.

Part 1 tells you the purpose of this study and what will happen to your child if you take part. Part 2 gives you more detailed information about the conduct of the study.

PART 1

1. What is the purpose of the study?
We aim to find out the effectiveness of the ketogenic diet (KD) in reducing seizures, compared to the use of further medicines that are used to help seizures (anti-epileptic drugs or AEDs) in children aged 3 months to 2 years, who have failed to respond to two or more AEDs.

The ketogenic diet has been shown to be successful in controlling seizures in many observational studies, and in a randomised trial in older children (aged 2 to 16 years). We wish to determine if this is the case in younger children.

The ketogenic diet is a low carbohydrate and high fat diet designed to mimic the effects of starvation on the body. It is carefully planned to provide sufficient energy and protein to maintain growth and development. The basis of the diet is that the main energy source is fat, which is used in the body and produces molecules called ketones.

We will also examine the possible role of a component of the diet, medium chain fatty acids, in giving the antiepileptic effect. The study would be the first of its kind in children under two years of age, and would make a significant contribution to the evidence-base for treatment of infants with epilepsy.

2. Why has my child been invited?
Your child has been invited to take part in this study because they have ongoing epileptic seizures despite treatment with 2 or more antiepileptic drugs. We aim to include 160 children across the UK to take part in this study.

3. Does my child have to take part?
No. The decision for you and your child to take part in the study is entirely voluntary and you may refuse to take part without giving a reason. We will describe the study and go through this information sheet. If you agree to take part, we will then ask you to sign a consent form. You are free to withdraw at any time without giving a reason. This would not affect the standard of care your child receives.

4. What will happen to my child if they take part?

If your child is eligible to enter the study, he/she will be assigned randomly to one of two groups. This is a randomised trial as sometimes we don't know which way of treating patients is best. To find out, we need to compare different treatments. We put people into groups and give each group a different treatment. The results are compared to see if one is better. To try to make sure the groups are the same to start with, each patient is put into a group by chance (randomly).

One group will receive a further antiepileptic drug treatment as per routine practice and the other group will follow the ketogenic diet for 8 weeks.

We put each patient into a group randomly because we want to prevent selection bias by using the play of chance to assign participants. This means that the decision on which group your child is assigned to will not be related to their diagnosis or responsiveness to treatment, but to chance. As one group will receive intervention (ketogenic diet) calculated by a dietician, to ensure fair statistical comparison with a group with no dietician, slightly more children will be randomised to receive the diet (92) than a further AED (68). If your child is randomised to receive a further AED and their seizures continue, you will be offered the possibility of initiating the ketogenic diet after the 8-week period if their seizures are no better.

Whichever group your child is in, they will be in the study for a total of 12 months. There will be a maximum of 9 study visits: screening, baseline and randomisation, at 4 weeks, 8 weeks and at 3, 6, 9, and 12 months. Most of the assessments and procedures are carried out as routine care; however, a few extra assessments as part of the research study will be undertaken. Please note that not all procedures will be carried out at all study visits and that a couple of extra visits may be required. After 8 weeks if your child has received a further AED and seizures continue, you will be offered the possibility of initiating the ketogenic diet. Please see the flow chart on page 8, which shows a summary of the study.

Assessments

- **Physical examination:** children in both groups will have a full examination to check their physical condition including weight, length, head circumference, blood pressure, pulse, and temperature. Also you will be asked to provide information about your child's general health, medical history and the medications they take.

- **Blood tests:** children in both groups will also have a blood test to ensure that mineral and supplement levels are appropriate and to make sure that their liver, kidneys and blood lipid levels are normal. These will be re-checked at 8 weeks, and should your child be on a ketogenic diet 6 monthly thereafter.

Assessments as part of this study

- **Dietitian visit and training:** parents will be asked to complete a 3 day food diary based on which the dietitian will calculate your child's ketogenic diet. Training will be given so that the diet can be followed at home as per standard practice in your hospital.

- **Ketogenic diet:** children in the ketogenic diet group will be asked to follow the prescribed ketogenic diet for 8 weeks. During that period their ketone levels should be monitored twice daily and recorded in the seizure diary.

- **Questionnaires:** during study visits you will be asked to complete questionnaires to describe how your child is feeling.

- **Seizure diary:** you will be asked to complete a seizure diary for your child daily between visits.

- **Blood samples:** to evaluate chemicals in the blood and assess whether medium chain fatty acids are associated with seizure control. This is only for patients on the ketogenic diet.

- **Adverse events:** the study team will ask you about any side effects or problems your child is experiencing.

- **Home monitoring:** During the ketogenic diet the level of ketones in your child's urine (dipstick) or blood (finger prick) should be tested and recorded, twice daily. We will provide home monitoring kits and training on how to use these kits.

The assessments are shown in a table on page 9 of the information sheet.

If randomised to receive the ketogenic diet a dietitian will provide constant advice and guidance. The ketogenic diet can be demanding and restrictive in order to provide the desired results, as you will need to follow specific meal plans desiged for your child, aiming to finish the prescribed intake of food. Prior to the start you will have to complete a food diary for 3 days (including 1 weekend day). Based on the information provided the dietitian will calculate the diet to suit your child's needs and will provide instructions regarding meal planning and allowed/prohibited foods.

The ketogenic diet will be the classical version which is calculated in a ratio of ketone-producing foods (fat) to those which reduce ketone production (carbohydrate and protein). Foods appropriate to the age of your child will be included, however carbohydrate-rich foods are not allowed, such as bread, pasta, breakfast cereals, sweets and some fruits and vegetables like bananas and potatoes. Typical foods included in the diet are meat, fish, eggs and cheese (protein sources), low-carbohydrate fruit and vegetables such as dark-leafy greens, berry fruits and avocados, and a fat source at each meal, for example, cream, butter or oil. A prepared ketogenic formula feed called Ketocal will usually be included in the diet; this is available on prescription. If your child is still being breast fed, feeding can be continued on the ketogenic diet, in combination with the ketogenic feed which will be given in a prescribed amount before each breastfeed. If breast milk is expressed, this can be mixed with the ketogenic feed to the correct ketogenic ratio. Infants on a ketogenic diet can be weaned as normal; the dietitian will give advice on how to adapt standard weaning foods to the correct ketogenic ratio by adding extra fat.

Training about carrying out the diet at home will be provided. The diet should be followed for 8 weeks, after which a decision will be made in consultation with your neurologist and dietitian if your child should continue on the diet, if it is proving effective for seizure control. During the diet the level of ketones in your child's urine (dipstick) or blood (finger prick) should be tested and recorded, twice daily. After 8 weeks if your child does not improve on the ketogenic diet, your doctor will change back to standard medical practice (AEDs).

If randomised to the anti-epileptic drug group, your child will receive the further anti-epileptic medicines as decided by your doctor. After 8 weeks if your child has received a further AED and seizures continue, you will be offered the possibility of initiating the ketogenic diet.

5. What are the alternatives for diagnosis or treatment?

Your child has not responded to at least 2 antiepileptic drugs. Both a further antiepileptic drug treatment and the ketogenic diet that are part of this study are commonly provided in your hospital as standard treatment for children with complex epilepsy.

If your child takes part in the study, treatment with the usual medication will continue. Your child's doctor will remain free to give alternative treatment to that specified in the study, at any stage if it is deemed to be in the best interest of your child.

If your child is not eligible to take part in the study or you decide not to participate or to withdraw, the treatment to be received will be discussed with the child's doctor and will depend upon the policy of your child's hospital.

6. What are the possible disadvantages and risks of taking part?
Before you decide for your child to take part in the study we would like to inform you of all possible risks of taking part so you can make a fully informed decision:

- A finger-prick test used to check for the presence of ketones in blood may cause temporary pain and soreness.
- When blood samples are taken your child might experience some temporary discomfort and bruising at the site of needle entry may occur. If required, anaesthetic cream before sample collection will be used.
- Possible side effects of the ketogenic diet are explained below

Before deciding to take part, you should also consider if taking part in the study will affect any private medical insurance you have and seek advice from your insurance company if necessary.

7. What are the side effects of any treatment received when taking part?
The change to a new diet as well as the restrictive nature on the ketogenic diet can cause a number of side effects, which can usually be minimised by making minor adjustments to the diet.

From previous research we have found the following effects:

Common side effects may include: lethargy (initial stage) and acidosis, constipation, change in lipid (fat) levels in the blood, diarrhoea, hunger, vomiting, increase in blood uric acid levels.

Occasional side effects may include: renal stones, hypoglycaemia.

Rare side effects may include: pancreatitis, bruising, vitamin and/or mineral deficiency, abdominal pain, gallstone formation, dehydration.

We will monitor for possible side effects during the study by asking you to complete a ketogenic diet side effects questionnaire at the start of the diet, and at 4 and 8 weeks afterwards. We will also give you training and look at the results of the home monitoring urine and blood finger prick tests to check for abnormal blood and urine levels. Your child will be clinically monitored for side effects at each clinic visit and regular blood samples will help us to check the presence of unwanted side effects.

8. What are the possible benefits of taking part?
The ketogenic diet is known to reduce the frequency of seizures in children with drug resistant epilepsy; however, there is no guarantee that being in the study will help your child. Ultimately, results of this study may help to optimise future standard treatment in children with drug resistant epilepsy.

9. What happens when the research study stops?
The study stops when all the children have completed their interventions and follow-up visits or if the study has to be closed prematurely for any other reason. Your child's doctor will arrange for their care to continue as per standard practice. Your hospital offers antiepileptic drug therapy and ketogenic diet services as standard treatment for complex paediatric epilepsy; therefore, those services will also be available after the end of the study after a consultation with your child's doctor.

10. What if there is a problem?
Any complaint about the way you have been dealt with during the clinical trial or any possible harm you might suffer will be addressed. The detailed information concerning this is given in Part 2 of this information sheet. If you have any concerns or complaints you should contact your study doctor in the first instance.

11. Will my taking part in the study be kept confidential?
Yes. We will follow ethical and legal practice and all information about you will be handled in confidence. The details are included in Part 2.

12. Expenses and payments
Participation in the study is voluntary and no payment is to be received. The follow-up and study visits will be planned to coincide with routine care visits, therefore, no additional travel expenses are expected. In the case that you and your child are required to attend extra study visits reasonable travel expenses will be reimbursed upon production of valid receipts (i.e. petrol, train tickets).

13. Contact Details

Further details: Please contact your doctor or another study team member on the telephone number given below if you have any queries about the study or you would like to discuss your child's participation in the study. Should your child have to visit another doctor tell him/ her that they are taking part in this study so that he/ she can contact your study doctor if necessary.

Local Investigator: xxxxxxxxxxxxxxxxxxxxxxxxx
Local Dietitian: xxxxxxxxxxxxxxxxxxxxxxxxx
This completes Part 1 of the Information Sheet.

If the information in Part 1 has interested you and you are considering participation, please read the additional information in Part 2 before making any decision.

PART 2

14. What if relevant new information becomes available?
Sometimes we get new information about the treatment being studied. If this happens, your research doctor will tell you about it and discuss with you whether you want to continue in the study. If you decide to withdraw your research doctor will make arrangements for the care of your child to continue. If you decide to continue in the study you will be asked to sign an updated consent form. Also, on receiving new information your research doctor might consider it to be in your best interests to withdraw your child from the study treatment. He / she will explain the reasons and arrange for your care to continue.

If the study is stopped for any other reason, we will tell you why and arrange your continuing care.

15. What will happen if I don't want to carry on with the study?
You can withdraw your child from the study at any time but we advise you to keep in contact with us to let us know your progress. Information collected up to that point will still be used. Any stored blood or tissue samples that can still be identified as yours will be destroyed if you wish.

16. What if there is a problem?

If you have a concern about any aspect of this study, you should ask to speak to the researchers who will do their best to answer your questions

If you remain unhappy and wish to complain formally, you can do this by contacting your local NHS Patient Advice and Liaison Service, known as PALS.

Every care will be taken in the course of this clinical trial. However in the unlikely event that you are injured by taking part, compensation may be available.

If you suspect that the injury is the result of the Sponsor's (University College London) or the hospital's negligence then you may be able to claim compensation. After discussing with your study doctor, please make the claim in writing to Professor Helen Cross who is the Chief Investigator for the clinical trial and is based at the Neurosciences Unit, UCL Institute of Child Health, 4/5 Long Yard, London WC1N 3LU; telephone number: 0207 5994105; fax number: 0207 430 0032; email address: h.cross@ucl.ac.uk. The Chief Investigator will then pass the claim to the Sponsor's Insurers, via the Sponsor's office. You may have to bear the costs of the legal action initially, and you should consult a lawyer about this.

Participants may also be able to claim compensation for injury caused by participation in this clinical trial without the need to prove negligence on the part of University College London (UCL) or another party. You should discuss this possibility with your study doctor in the same way as above.

Regardless of this, if you wish to complain, or have any concerns about any aspect of the way you have been approached or treated by members of staff or about any side effects (adverse events) you may have experienced due to your participation in the clinical trial, the normal National Health Service complaints mechanisms are available to you. Please ask your study doctor if you would like more information on this. Details can also be obtained from the Department of Health website: http://www.dh.gov.uk.

17. Will my taking part in this study be kept confidential?

If you consent to your child taking part in this study, the records obtained while your child is in this study as well as related health records will remain strictly confidential at all times. The information will be held securely on paper and electronically at your treating hospital and at UCL-Institute of Child Health, the main site managing this research under the provisions of the 1998 Data Protection Act. Your child's name and personal details will not be passed to anyone else outside the research team or the Sponsor (UCL). Your child will be allocated a trial number, which will be used as a code to identify you on all trial forms. Any information about your child which leaves the hospital will have their name, address and any personal data removed so that you cannot be recognised (if it is applicable to your research).

Your records will be available to people authorised to work on the trial but may also need to be made available to people authorised by the Sponsor, which is the organisation responsible for ensuring that the study is carried out correctly. By signing the consent form you agree to this access for the current study and any further research that may be conducted in relation to it, even if you withdraw from the current study.

The information collected about you may also be shown to authorised people from the UK Regulatory Authority (the Medicines and Healthcare Products Regulatory Authority); this is to ensure that the study is carried out to the highest possible scientific standards. All will have a duty of confidentiality to you as a research participant.

If you withdraw consent from further study treatment, unless you object, your data and samples will remain on file and will be included in the final study analysis.

In line with the regulations, at the end of the study your data will be securely archived for a minimum of 20 years. Arrangements for confidential destruction will then be made.

18. Will my GP be informed of my involvement?

With your permission, your GP will be notified that you and your child are taking part in this study.
No information will be released without your consent.

19. What will happen to any samples I give?

Your child's blood sample will be analysed by the Clinician Scientist based at UCL - Institute of Child Health, who is part of the research team. All information collected including seizure data, ketone levels and neurodevelopmental evaluation will be held on a computer server at UCL and only Professor Cross and the research team undertaking this study will have access to it. At the end of the study the samples will be stored for 20 years maintaining confidentiality to uphold good research practice. All samples given to us will be considered a gift. We will only use these samples for this study and they will be stored and disposed of securely.

20. What will happen to the results of the research study?

The results of the study will be available after it finishes and will be published in a medical journal as well as presented at scientific conferences. The data will be anonymous and none of the patients involved in the trial will be identified in any report or publication.

Should you wish to see the results, or the publication, please ask your study doctor.

21. Who is organising and funding the research?

The research is being organised through UCL-Institute of Child Health in collaboration with Great Ormond Street Hospital for Children NHS Trust, Evelina Children's Hospital, Alder Hey Children's Hospital, Royal Manchester Children's Hospital, Addenbrookes NHS Trust, Birmingham Children's Hospital, Leeds Teaching Hospital NHS Trust, University of Bristol, Matthew's Friends Clinics and Young Epilepsy. It is funded by the National Institute for Health Research and sponsored by UCL.

22. Who has reviewed the study?

All research in the NHS is looked at by an independent group of people, called a Research Ethics Committee, to protect you and your child's interests. This study has been reviewed and given favourable opinion by London Fulham Research Ethics Committee.

23. Further information and contact details

You are encouraged to ask any questions you wish, before, during or after your treatment. If you have any questions about the study, please speak to your study nurse or doctor, who will be able to provide you with up to date information about the procedure(s) involved. If you wish to read the research on which this study is based, please ask your study nurse or doctor. If you require any further information or have any concerns while taking part in the study please contact one of the following people:

Local Investigator: xxxxxxxxxxxxxxxxxxxxxxxx
Local Dietitian: xxxxxxxxxxxxxxxxxxxxxxxx

Alternatively if you or your relatives would like general information about research please review the UKCRN website: www.UKCRN.org.uk

If you decide you would like to take part then please read and sign the consent form. You will be given a copy of this information sheet and the consent form to keep. A copy of the

consent form will be filed in your patient notes, one will be filed with the study records and one may be sent to the Research Sponsor.

You can have more time to think this over if you are at all unsure.

Thank you for taking the time to read this information sheet and to consider this study.

Appendix 3: GP letter v1.0 dated 17/06/2014

Department: 0207 188 3998
Fax: 0207 188 4629
Main Switchboard: 020 7188 7188

Date:

Dear Dr.............

Re: Patient name..

Ketogenic diet treatment of epilepsy in infants

The above-named patient from your practice has consented to enter the above study being carried out at xxxxxxxxxxxxxxxxxxx in collaboration with UCL – Institute of Child Health.

The parent/guardian has been given an information sheet (a copy of the parent/guardian information leaflet is attached) and is aware that he/she/their child can withdraw from the study at any time without giving a reason.

Follow-up will be organised by the research team and should not entail any additional workload for you.

Please do not hesitate to contact me if you require any further details about this study.

With best wishes

<Local PI contact>

Department

Professor Helen Cross
Prince of Wales's Chair of Childhood
UCL-Institute of Child Health
4/5 Long Yard
London
Telephone number: 0207 5994107
Email:h.cross@ucl.ac.uk
