# Supplementary material for: Ketogenic diet in the treatment of epilepsy in children under the age of 2 years: study protocol for a randomised controlled trial
Source: Trials. 2017 Apr 26;18:195. doi: 10.1186/s13063-017-1918-3 (PMC5406967; doi:10.1186/s13063-017-1918-3)
Supplement: Supplementary file 4 — (AED consensus flowchart). (DOCX 44 kb) [file 13063_2017_1918_MOESM4_ESM.docx]

**Additional file 4. (AED consensus flowchart)**

GTC/tonic:

Assume VPA/LEV

Absence:

Assume VPA/LEV

Generalised

TPM

LEV, TPM, VPA, NTZ

Not Dravet:

Select on main seizure type

Dravet:

Assume

V PA, CLB, STP

Not spasms

Onset of epilepsy <24m

Focal: assume CBZ /LEV

TPM

TPM

Spasms: assume had VGB/steroids

| Carbamazepine (Tegratol)  Clobazam (Frisium)  Clonazepam (Rivotril)  Ethosuximide (Zarontin)  Lacosmide (Vimpat)  Lamotrigine (Lamictal)  Levetiracetam (Keppra)  Nitrazepam (Mogadon)  Phenytoin (Epanutin)  Rufinamide (Inovelon)  Sodium Valproate (Epilim)  Stiripentol (Diacomit)  Topiramate (Topamax)  Vigabatrin (Sabril)  Zonisamide (Zonegran) |
| --- |

ETX
