# Supplementary material for: Ketogenic diet in the treatment of epilepsy in children under the age of 2 years: study protocol for a randomised controlled trial
Source: Trials. 2017 Apr 26;18:195. doi: 10.1186/s13063-017-1918-3 (PMC5406967; doi:10.1186/s13063-017-1918-3)
Supplement: Supplementary file 5 — Names and descriptions of investigational medicinal products used in KIWE. (DOCX 15 kb) [file 13063_2017_1918_MOESM5_ESM.docx]

**Additional file 5. Names and descriptions of investigational medicinal products used in KIWE**

| **Medicines** | **Formulation** | **Concentration** |
| --- | --- | --- |
| Carbamazepine (Tegratol) | Liquid | 100mg/5mL |
|  |  |  |
| Clobazam (Frisium) | Oral suspension | 5mg/5mL |
|  |  | 10mg/5mL |
|  |  |  |
| Clonazepam (Rivotril) | Oral solution | 0.5mg/5mL |
|  |  | 2mg/5mL |
|  | Oral drops | 2.5mg/mL |
|  |  |  |
| Ethosuximide (Zarontin) | Syrup | 250mg/5mL |
|  |  |  |
| Lacosmide (Vimpat) | Syrup | 10mg/mL |
|  |  |  |
| Lamotrigine (Lamictal) | Dispersible tablets | 2mg |
|  |  | 5mg |
|  |  | 25mg |
|  |  | 100mg |
| Levetiracetam (Keppra) | Oral solution | 100mg/mL |
|  |  |  |
| Nitrazepam (Mogadon) | Oral suspension | 2.5mg/5mL |
|  |  |  |
| Phenytoin (Epanutin) | Suspension | 30mg/5mL |
|  | Infatabs | 50mg |
|  | Capsules | 25, 50, 100mg |
| Rufinamide (Inovelon) |  |  |
|  | Oral suspension | 40mg/mL |
| Sodium Valproate (Epilim) | Oral solution | 200mg/5mL |
| Stiripentol (Diacomit) | Powder (sachets) | 250mg |
|  |  | 500mg |
|  |  |  |
| Topiramate (Topamax) | Sprinkle capsules | 15mg |
|  |  | 25mg |
|  |  | 50mg |
|  |  |  |
|  |  |  |
|  |  |  |
| Vigabatrin (Sabril) | Powder (Sachets) | 500mg |
| Zonisamide (Zonegran) | Capsules | 25mg |
|  |  | 50mg |
|  |  | 100mg |
